# A Disposable Alkaline Phosphatase-Based Biosensor for Vanadium Chronoamperometric Determination

**DOI:** 10.3390/s140203756

**Published:** 2014-02-24

**Authors:** Ana Lorena Alvarado-Gámez, María Asunción Alonso-Lomillo, Olga Domínguez-Renedo, María Julia Arcos-Martínez

**Affiliations:** 1 CELEQ and School of Chemistry, University of Costa Rica, San Pedro de Montes de Oca, San José P.O. Box 11500-2060, Costa Rica; 2 Department of Chemistry, Faculty of Sciences, University of Burgos, Plaza Misael Bañuelos s/n, Burgos 09001, Spain; E-Mails: malomillo@ubu.es (M.A.A.-L.); olgado@ubu.es (O.D.-R.); jarcos@ubu.es (M.J.A.-M.)

**Keywords:** alkaline phosphatase, biosensor, gold nanoparticles, 4-nitrophenyl phosphate, screen printed electrode, vanadium, water analysis

## Abstract

A chronoamperometric method for vanadium ion determination, based on the inhibition of the enzyme alkaline phosphatase, is reported. Screen-printed carbon electrodes modified with gold nanoparticles were used as transducers for the immobilization of the enzyme. The enzymatic activity over 4-nitrophenyl phosphate sodium salt is affected by vanadium ions, which results in a decrease in the chronoamperometric current registered. The developed method has a detection limit of 0.39 ± 0.06 μM, a repeatability of 7.7% (*n* = 4) and a reproducibility of 8% (*n* = 3). A study of the possible interferences shows that the presence of Mo(VI), Cr(III), Ca(II) and W(VI), may affect vanadium determination at concentration higher than 1.0 mM. The method was successfully applied to the determination of vanadium in spiked tap water.

## 1. Introduction

Being considered as one of the important transition elements in biological systems, vanadium is an ultra-trace metal that can be found in some marine organisms, in the prosthetic group of bromoperoxidases in certain marine algae [1–3], as part of the nitrogenase system of some bacteria and plants [4,5], as well as in plasma and inside cells of mammals [6]. It participates in the synthesis of chlorophyll in photosynthetic organisms and is a micronutrient of marine and terrestrial species [7].

In the past, vanadium compounds were used as a therapeutic agent for diabetes, anemia, chlorosis, and even for tuberculosis. It is also a tonic, antiseptic and as a spirocheticide. Nevertheless vanadium, especially as vanadium pentoxide, has a broad spectrum of known toxic effects on the respiratory, circulatory and central nervous systems, digestive organs, kidneys and skin in humans. However confirmative mutagenicity and carcinogenicity studies are not consistent, though they should be given priority in long exposure studies [8,9]. In recent years, vanadium has been used in the development of novel materials in biochemistry and industrial processes [10–12]. Its metallic form is used as a carbide stabilizer in making steels. Vanadium pentoxide is used in ceramics, as a catalyst, and in the production of superconductive magnets, and vanadyl sulfate and sodium metavanadate have been used in dietary supplements [8].

Industries using fossil fuels like petroleum, coal and oil, cause most of the discharges of vanadium into the environment. Mining areas are other sources of this contamination, while distillation and purification of crude oils contribute less vanadium into the atmosphere [13].

Vanadate in aqueous solution influences numerous enzyme-catalyzed reactions. Its effects on living systems and the different responses to the influence of vanadium are well documented [14]. As it can assume many stable anionic forms in aqueous solution, depending on acidity and concentration [15], it has been described as an inhibitor of different enzymes. Lindquist in 1973 [16] described the inhibition of ribonuclease by vanadate in the presence of uridine, explaining in some way the origin of the biological influences of vanadium compounds. A year later, in 1974, Van Etten and coworkers [17], demonstrated the influence of vanadate, molybdate and tungstate on phosphohydrolases such as acid phosphatases which are relatively nonspecific enzymes that catalyze the hydrolysis of several alkyl and aryl phosphate esters at a pH between 4 and 6. Lopez *et al.* showed that alkaline phosphatase, which is a metalloproteinase, catalyzes the hydrolysis of a number of phosphate esters, and there are a few competitive inhibitors of alkaline phosphatase aside from inorganic phosphate and arsenate, such as oxovanadium (IV) VO_2_^+^. It is also possible that vanadium (V) might adopt a trigonal bipyramidal structure since crystalline hydrated metavanadates (VO_3_^−^·H_2_O) are five-coordinate with oxygen atoms, and the geometry is approximately trigonal bipyramidal like phosphate, which is one of the reasons why vanadate is a known inhibitor (and sometimes stimulator) of many phosphate-metabolizing enzymes [18]. This includes the inhibition of a regulatory protein phosphatase, which is likely to lead to activation of a protein kinase, the activity of which is key to the insulin-mimetic action of vanadate [17,18]. It also can inhibit hexokinase, adenylate kinase and phosphofructokinase [15]. Vanadate-dependent haloperoxidases have been shown to attain phosphatase activity, and this finding may have some impact on medical applications. Another important impetus to vanadium coordination chemistry has arisen from the observation that vanadate, peroxovanadate, vanadyl and several vanadium complexes exert an insulin-mimetic effect [6].

Electrochemical biosensors based on the principle of enzyme inhibition have been applied for a wide range of toxic analytes such as pesticides, derivatives of insecticides, heavy metals and glycoalkaloids [19]. Because of their excellent performance capabilities, such as rapid response, high specificity and sensitivity, relatively compact size, low cost and easy operation, these biosensors can be a good alternative for the detection of vanadium [20].

Alkaline phosphatases (ALPs), which catalyze the hydrolysis of phosphate esters, are widely distributed in mammalian tissues, and are present in high concentrations in bones, intestines, kidneys, placenta, and liver [21]. ALP is probably the most commonly used conjugated enzyme for immunoassays due to its high turnover number, broad substrate specificity and possibility of application. The determination of its activity has been carried out using various spectrophotometric and electrochemical methods [21–25]. In the development of sensitive electrochemical ALP-based assays stable substrates such as phenyl phosphate [26–28], naphthyl phosphate [28,29], ascorbic acid 2-phosphate [28,30], *p*-nitrophenyl phosphate [28,31] and rivoflavin-5-monophosphate [20] have been used. Among them, *p*-nitrophenyl phosphate is probably one of the most widely used substrate for ALP, since the enzymatically produced *p*-nitrophenol can be detected electrochemically [31].

Reversible inhibition of ALP by vanadium has been previously reported [18,20,25], although this interaction has been scarcely used for vanadium determination [20]. The presence of vanadium produces a decrease of the chronoamperometric reduction signal registered that can be related to the concentration of this species.

Thus, the aim of this work has been the development of a screen-printed based amperometric biosensor, easily usable in any analytical laboratory, for the detection of vanadium. ALP has been cross-linked to the working electrode of screen-printed carbon electrodes (SPCEs) previously modified by gold nanoparticles (ALP-AuNPs-SPCEs). In order to obtain a biosensor with improved conductivity and performance for vanadium detection, AuNPs were deposited onto the working electrode previous to the enzyme immobilization [32]. The use of AuNPs have been reported in order to enhance the chronoamperometric current response, yielding a sensor with an excellent electrocatalytic response, fast response time, long term stability and reproducibility [32–38]. The ALP-based biosensor has been characterized for the detection of vanadium in water samples. Figures of merit, such as precision or limit of detection, have been evaluated.

## 2. Results and Discussion

In a previous paper 5-riboflavin monophosphate was used as a substrate for an alkaline phosphatase biosensor because there was no report of such a substrate being used for a biosensor. Preechaworapun [28] presented a list of the substrates for this type of enzymes, and Fanjul [31] studied the detection of *p*-nitrophenol in alkaline phosphatase assays. In our case we proved several substrates recommended by Preechaworapun such as 3-indoxyphenyl phosphate, 1-naftyl phosphate and *p*-nitrophenyl phosphate, but the last one presented higher currents, and also ALP inhibition currents decreased significantly with vanadium additions.

As mentioned above, *p*-nitrophenyl phosphate is hydrolyzed by ALPs, under alkaline conditions, to 4-nitrophenol, which is electrochemically oxidized, originating a well-defined oxidation current [31]. Taking into account that the enzymatic activity of ALPs is inhibited by vanadium [18,20,25], the presence of this metal into the electrochemical cell results in a current decrease. In this way, the difference between the steady-state current in the absence of vanadium (I_0_) and the steady-state current in the presence of vanadium (I) (ΔI (I_0_–I)) can be quantitatively related to concentration of vanadium added.

In order to quantify this kind of electrochemical current, an ALP-based biosensor was built according to the procedure described in Section 3.3. This chronoamperometric current depends on experimental factors, such as pH of supporting electrolyte, substrate concentration, working potential or ionic strength of the medium (concentration of Cl^−^ ions into the electrochemical cell). In order to maximize the registered inhibition current, the effect of these variables and their interactions in the chronoamperometric response was at first evaluated by the experimental design methodology [39–41]. The experimental domain was defined by the values shown in Table 1, corresponding to the high (+) and low (−) levels for each factor. Then, the 17 experiments corresponding to all those possible combinations, bearing in mind the three replicates in the central point necessary to estimate the residual value, were carried out. Once the oxidation current registered due to the enzymatically produced *p*-nitrophenol was stable, vanadium was added, quantifying the chronoamperometric current of a 1.8 μM solution as response variable for the analysis.

**Figure 1.**
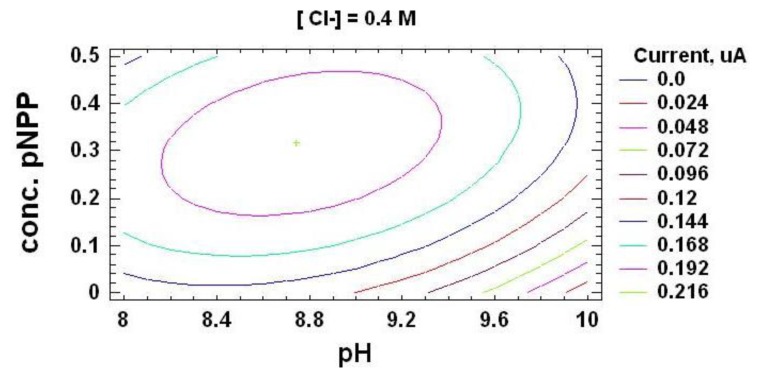
Contours of estimated response surface for alkaline phosphatase and *p*-nitrophenyl phosphate at [Cl^−^] = 0.40 M. The central green point in the center of the circle is considered the optimum values for experimental variables mentioned above.

From this optimization process, the following optimum values for the experimental variables in the vanadium determination were used: supporting electrolyte pH 8.7, working potential of + 0.8 V *vs.* Ag/AgCl SPE, substrate concentration of 0.32 mM and Cl^−^ concentration of 0.36 M. Easily quantifiable chronoamperometric signals are registered under these optimized conditions for vanadium (Figure 1).

**Figure 2.**
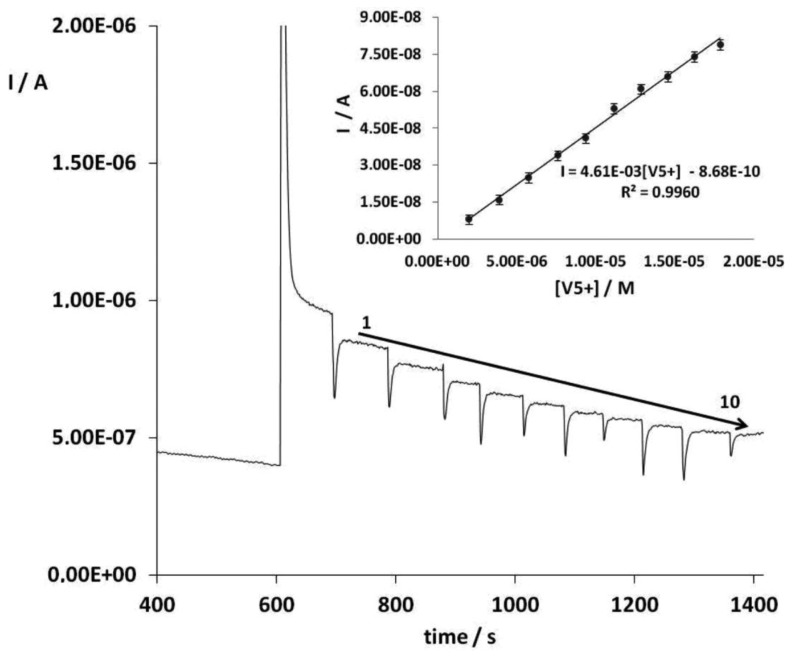
Chronoamperogram registered using an ALP-based biosensor under the optimum conditions (applied potential, + 0.80 V *vs.* Ag/AgCl SPE; supporting electrolyte pH 8.7 (Tris buffer, 0.36 M total Cl^−^) and *p*-nitrophenyl phosphate, 0.32 mM) in the vanadium concentration range from 3.0 μM to 30.0 μM. Insert figure, a calibration curve for vanadium additions at the optimum conditions.

Control experiments were carried out under the optimum conditions using bare SPCEs and AuNPs-SPCEs but without enzyme as reported in Section 3.3. No analytical signal was obtained, that is to say, the inhibition response registered after the addition of the substrate is only related to vanadium concentration. Therefore, vanadium can be determined by its inhibitory effect on the response of ALP to *p*-nitrophenyl phosphate. Figure 2 shows the amperometric signals of the substrate addition and vanadium additions 1 to 10 under optimal conditions. An insert figure shows a calibration curve for vanadium V.

The inhibitory effect of this metal in the enzymatic activity, when using *p*-nitrophenyl phosphate as substrate, was also studied by the kinetic parameters of the Lineweaver-Burk plot (V_max_ and K_m_), in absence and presence of vanadium. In absence of the metal, V_max_ and K_m_ were 1.1 × 10^−6^ and 2.8 × 10^−4^, respectively. In presence of vanadium, it was observed that V_max_ and K_m_ increased: with 3.8 μM of vanadium, V_max_ = 2.1 × 10^−6^, K_m_ = 6.5 × 10^−4^, and with 11 μM of vanadium V_max_ = 2.5 × 10^−6^ M and K_m_ = 9.9 × 10^−4^ which suggest a mixed inhibition [42]. Thus, the inhibitory effect of vanadium on the ALP/*p*-nitrophenyl phosphate reaction has been confirmed for the higher affinity of ALP for *p*-nitrophenyl phosphate in the absence of this metal.

The detection of vanadium through the inhibition of ALP/*p*-nitrophenyl reaction (Calibration range from 0.8 μM to 30.0 μM) has resulted more sensitive than the reported one based on the inhibition of ALP/riboflavin-5-monophosphatase (calibration range from 1.8 μM to 15.0 μM) [15]. In this way, the limit of detection based on the standard deviation (Sy/x) of the responses for the blank injection in triplicate and the slope of the calibration curve was 0.39 ± 0.07 μM, one order lower than the previously one reported [20].

Precision of the developed procedure was studied in terms of repeatability (intra-biosensor) and reproducibility (inter-biosensors). Both figures of merit have been determined as the relative standard deviation (RSD) of the slopes of four calibration curves built under the optimum conditions of the experimental variables. Values of 7.7% and 8% (n = 4) were obtained for repeatability and reproducibility, respectively.

The performance of the developed procedure was checked by its accuracy and trueness. The accuracy of the proposed method was evaluated by means of the analysis of a vanadium certified sample (High Purity Standards(R) Vanadium Standard solution with a Certificate of Analysis confirmed against SRM 3165, lot 992706, certified value (1,000 ± 4) mg L^−1^). The vanadium mean concentration quantified, 1,055 ± 65 mg L^−1^ (n = 4; α = 0.05), matches the certified value of the sample. The method also showed a satisfactory value for trueness, evaluated by recovery studies, since the added vanadium concentration values (7.33 μM) were in good agreement with the found concentration value of 7.56 ± 0.14 μM (n = 4, α = 0.05). The average recovery for this analysis was 103.1 ± 3.6% with a RSD of 3.5%. Therefore, the proposed method is both accurate and suitable for the analysis of vanadium.

The possible interference from other metals, such as Ca(II), Sn(II), Al(III), Fe(III), Cr(III), As(V), Mo(VI) and W(VI), has been studied. Their effect was analyzed by measuring the inhibition current after consecutive additions of several solutions of each metal. It was observed that Al(III), As(V), Fe(III) and Sn(II) have a null influence on the vanadium chronoamperometric response. However, Cr(III), Mo(VI) and W(VI) present a higher inhibition current, so these metals must be taken into account in the analysis of vanadium.

Finally, the developed procedure was applied to the determination of vanadium in spiked tap water samples (1.96 μM), by standard addition methodology in quadruplicate. The concentration of vanadium found was 1.99 ± 0.23 μM (*n* = 4, α = 0.05, RSD = 6.8%), with an average recovery of 101%.

## 3. Experimental Section

### 3.1. Chemical Reagents

Several inks were used in the fabrication of the screen printed electrodes (SPEs), namely Electrodag PF-407 A (carbon ink), Electrodag 6037 SS (silver/silver chloride ink) and Electrodag 452 SS (dielectric ink) all supplied by Acheson Colloiden (Scheemda, The Netherlands). Analytical grade chemicals with no additional purification were used. All solutions were prepared in ultrapure water, conductivity of 0.05 μS/cm (Gen-Pure TKA, Niederelbert, Germany).

Hydrogen tetrachloroaurate (III) trihydrate (HAuCl_4_), ALP, bovine serum albumine (BSA) and glutaraldehyde (GA) were obtained from Sigma Chemical Co. (St. Louis, MO, USA). *p*-Nitrophenyl phosphate sodium salt was acquired from Fluka Analytical (Buchs, Switzerland). Ammonium metavanadate (Merck, Darmstadt, Germany) was used as stock solution of vanadium. 28 mM Tris(hydroxymethyl)aminomethane buffer (Aldrich Chemical Co., Buchs, Switzerland) was used together with 19 mM of MgCl_2_ (Merck) and 0.36 M total Cl^−^, (Merck) as supporting electrolyte. HCl (Merck) was used to adjust the pH value.

### 3.2. Apparatus

SPCEs were produced on a DEK 248 printing machine (DEK, Weymouth, UK) using polyester screens with appropriate stencil designs. Electrochemical measurements were made with an Autolab 128N electrochemical system with GPES software (Echo Chemie, Utrecht, The Netherlands). The pH measurements were performed using a Mettler-Toledo pHmeter S47-K (Columbus, OH, USA).

### 3.3. Manufacturing of ALP-AuNPs-SCPEs

**Figure 3.**
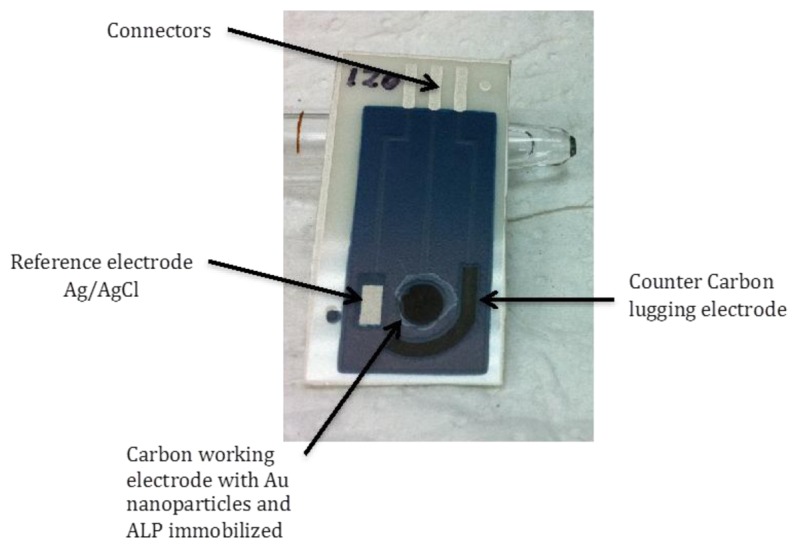
ALP-AuNPs-SPCE used for vanadium determination. Carbon working electrode area, 12.6 mm^2^.

SPCEs were produce by sequential layer deposition of each component, that is conductive silver tracks, Ag/AgCl reference electrode (Ag/AgCl SPE), carbon counter and working electrodes and finally, dielectric ink, according to the procedure described anywhere else [43]. The different inks were cured according to the manufacturer's specifications. Screen-printed configurations of three electrodes (working, reference and counter electrode) were thus obtained (Figure 3).

**Figure 4.**
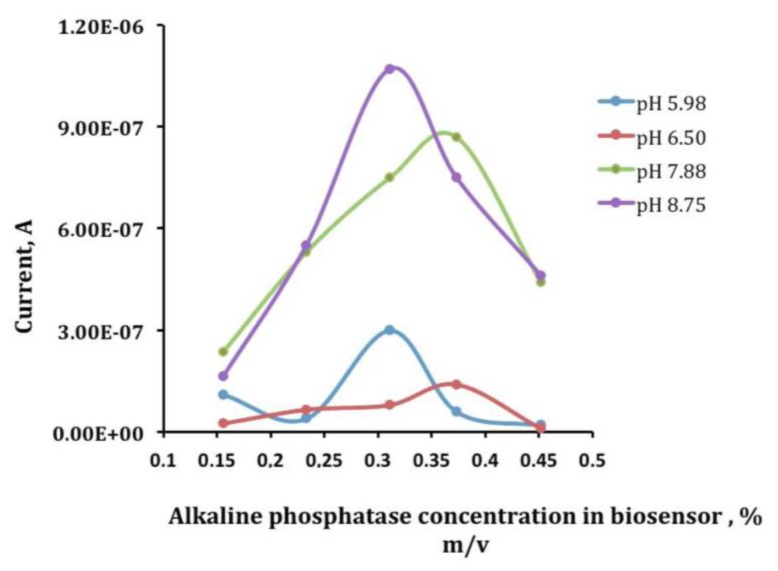
Currents obtained by p-NPP additions using electrodes with different ALP enzyme concentrations.

**Figure 5.**
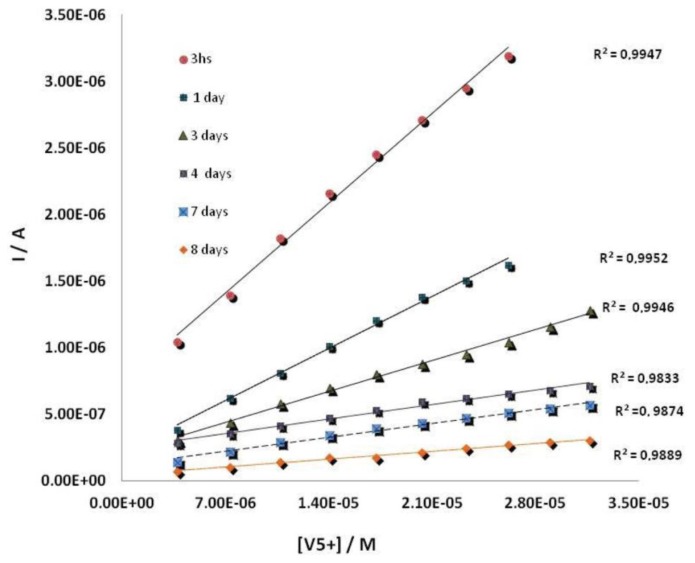
Calibration curves for ALP biosensor measured at different days from electrode preparation.

The working electrode of these devices was electrochemically modified by AuNPs, using a 0.1 mM solution of HAuCl_4_ in 0.5 M H_2_SO_4_. The deposition was performed by applying a potential of + 0.18 V (*vs.* Ag/AgCl SPE) during 15 s under stirring conditions [20,44]. The enzyme was then immobilized by cross-linking on the surface of AuNPs modified SPCEs. To optimize an appropriate mixture of ALP enzyme, BSA and GA, several electrodes with different quantities of the enzyme from 20 μL to 80 μL, 10 μL–20 μL BSA and 20 μL–40 μL glutaraldehyde, were prepared and the best results were obtained with a mixture made up of 40 μL of ALP 0.6%, 20 μL of BSA 1.75% (w/v) and 40 μL of GA, 2.5% (w/v) which gave the best current response (Figure 4) by dropping 10 μL of a 2:1:2 mixture of a 0.6% of enzyme solution, 1.75% (w/v) of BSA solution and 2.5% (w/v) of GA solution onto the surface of a screen-printed working electrode [20]. Finally, the mixture was left to react at 4 °C during 1 h. ALP-AuNPs-SPCEs were stored at 4 °C. Under these storage conditions the developed biosensor showed a good stability for at least one week. Figure 5 shows calibration curves for vanadium prepared the same day but measured at different times from electrode preparation.

### 3.4. Measurement Procedure

Chronoamperometric measurements were performed at room temperature in a cell containing 5 mL of supporting electrolyte solution, of the desired pH, under constant mechanical stirring. An ALP- based biosensor was placed in the electrochemical cell containing 5 mL of supporting electrolyte solution. An adequate potential was applied and, once a steady-state current was set, a defined amount of *p*-nitrophenyl phosphate stock solution was added to the measuring cell. A large anodic current was observed due to the oxidation of the enzymatically produced *p*-nitrophenol. Then, once a plateau corresponding to the steady-state response was reached again, fixed portions of the vanadium stock solution were added consecutively. Enzyme electrodes were conditioned in a buffer solution for 5 min between each calibration setting.

## 4. Conclusions

The use of ALP based biosensors using AuNPs/SPCEs allows the selective chronoamperometric determination of vanadium. This biosensor offers better figures of merit compared with the previous work [20] using 5-monophosphate, lower limit of detection, wider linear range, but the same interferences, W(VI) and Mo(VI), which are the most significant at μM levels. The effect of vanadium in the ALP/*p*-nitrophenyl phosphate reaction results in a mixed inhibition, which allows the quantification of vanadium in tap water. The developed procedure has shown a limit of detection of 0.39 ± 0.06 μM, ten times lower than previously reported. The reproducibility and repeatability values of RSD for the slopes of several calibrations are lower than 10%.

## Figures and Tables

**Table 1.** Values corresponding to the high (+) and low (−) levels for each factor used in the optimization of the experimental conditions for vanadium detection.

	**Low Level**	**High Level**
Supporting electrolyte pH	7.0	9.6
Working potential	+ 0.5 V *vs.* Ag/AgCl SPE	+ 1.0 V *vs.* Ag/AgCl SPE
Substrate concentration	0.13 mM	0.47 mM
Ionic strength	0.26 M	0.46 M

## References

[1] Michibata H., Yamaguchi N., Uyama T., Ueki T. (2003). Molecular biological approaches to the accumulation and reduction of vanadium by ascidians. Coordinat. Chem. Rev..

[2] Mukherjee B., Patra B., Mahapatra S., Banerjee P., Tiwari A., Chatterjee M. (2004). Vanadium—An element of atypical biological significance. Toxicol. Lett..

[3] Ueki T., Michibata H. (2011). Molecular mechanism of the transport and reduction pathway of vanadium in ascidians. Coordinat. Chem. Rev..

[4] Rehder D. (2000). Vanadium nitrogenase. J. Inorg. Biochem..

[5] Janas Z., Sobota P. (2005). Aryloxo and thiolato vanadium complexes as chemical models of the active site of vanadium nitrogenase. Coordinat. Chem. Rev..

[6] Rehder D. (1999). The coordination chemistry of vanadium as related to its biological functions. Coordinat. Chem. Rev..

[7] Rodríguez-Mercado J.J., Altamirano-Lozano M.A. (2010). Vanadio: Contaminación, metabolismo y genotoxicidad. Rev. Int. Contam. Ambient..

[8] (2012). Toxicological Profile For Vanadium U.S..

[9] (2000). Air Quality Guidelines for Europe; Vanadium, Chapter 6.12.

[10] Guidelli E.J., Guerra E.M., Mulato M. (2011). Ion sensing properties of vanadium/tungsten mixed oxides. Mater. Chem. Phys..

[11] Tsiafoulis C.G., Florou A.B., Trikalitis P.N., Bakas T., Prodromidis M.I. (2005). Electrochemical study of ferrocene intercalated vanadium pentoxide xerogel/polyvinyl alcohol composite films: Application in the development of amperometric biosensors. Electrochem. Commun..

[12] Zhu Z., Sun X., Wang Y., Zeng Y., Sun W., Huang X. (2010). Electrochemical horseradish peroxidase biosensor based on dextran–ionic liquid–V_2_O_5_ nanobelt composite material modified carbon ionic liquid electrode. Mater. Chem. Phys..

[13] Venkataraman B.V., Sudha S. (2005). Vanadium toxicity. Asian J. Exp. Sci..

[14] Bhattacharyya S., Tracey A.S. (2001). Vanadium (V) complexes in enzyme systems: aqueous chemistry, inhibition and molecular modeling in inhibitor design. J. Inorg. Biochem..

[15] Boyd D.W., Kustin K., Niwa M. (1985). Do vanadate polyanions inhibit phosphotransferase enzymes?. Biochim. Biophys. Acta.

[16] Lindquist R.N., Lynn J.L., Lienhard G.E. (1973). Possible transition-state analogs for ribonuclease. Complexes of uridine with oxovanadium (IV) ion and vanadium (V) ion. J. Am. Chem. Soc..

[17] Van Etten R.L., Waymack P.P., Rehkop D.M. (1974). Transition metal ion inhibition of enzyme-catalyzed phosphate ester displacement reactions. J. Am. Chem. Soc..

[18] Lopez V., Stevens T., Lindquist R.N. (1976). Vanadium ion inhibition of alkaline phosphatase-catalyzed phosphate ester hydrolysis. Arch. Biochem. Biophys..

[19] Amine A., Mohammadi H., Bourais I., Palleschi G. (2006). Enzyme inhibition-based biosensors for food safety and environmental monitoring. Biosens. Bioelectron..

[20] Alvarado-Gámez A.L., Alonso-Lomillo M.A., Domínguez-Renedo O., Arcos-Martínez M.J. (2013). Vanadium determination in water using alkaline phosphatase based screen-printed carbon electrodes modified with gold nanoparticles. J. Electroanal. Chem..

[21] Park J., Kim Y. (2013). An improved fluorogenic substrate for the detection of alkaline phosphatase activity. Bioorg. Med. Chem. Lett..

[22] Berezhetskyy A.L., Sosovska O.F., Durrieu C., Chovelon J.-M., Dzyadevych S.V., Tran-Minh C. (2008). Alkaline phosphatase conductometric biosensor for heavy-metal ions determination. ITBM-RBM.

[23] Szydłowska D., Campàs M., Marty J.-L., Trojanowicz M. (2006). Catechol monophosphate as a new substrate for screen-printed amperometric biosensors with immobilized phosphatases. Sens. Actuators B Chem..

[24] Zhang J., Cass A.E. (2000). Electrochemical analysis of immobilised chemical and genetic biotinylated alkaline phosphatase. Analy. Chimica Acta.

[25] Koncki R., Rudnicka K., Tymecki Ł. (2006). Flow injection system for potentiometric determination of alkaline phosphatase inhibitors. Analy. Chimica Acta.

[26] Ito S., Yamazaki S., Kano K., Ikeda T. (2000). Highly sensitive electrochemical detection of alkaline phosphatase. Analy. Chimica Acta.

[27] Serra B., Morales M.D., Reviejo A.J., Hall E.H., Pingarrón J.M. (2005). Rapid and highly sensitive electrochemical determination of alkaline phosphatase using a composite tyrosinase biosensor. Analy. Biochem..

[28] Preechaworapun A., Dai Z., Xiang Y., Chailapakul O., Wang J. (2008). Investigation of the enzyme hydrolysis products of the substrates of alkaline phosphatase in electrochemical immunosensing. Talanta.

[29] Abad-Villar E.M., Fernández-Abedul M.T., Costa-García A. (2002). Gold bands as a suitable surface for enzyme immunoassays. Biosens. Bioelectr..

[30] Kokado A., Arakawa H., Maeda M. (2000). New electrochemical assay of alkaline phosphatase using ascorbic acid 2-phosphate and its application to enzyme immunoassay. Analy. Chim. Acta.

[31] Fanjul-Bolado P., González-García M.B., Costa-García A. (2006). Flow screen-printed amperometric detection of p-nitrophenol in alkaline phosphatase-based assays. Analy. Bioanaly. Chem..

[32] González-García M.B., Costa-García A. (2000). Silver electrodeposition catalyzed by colloidal gold on carbon paste electrode: Application to biotin–streptavidin interaction monitoring. Biosens. Bioelectr..

[33] Siangproh W., Dungchai W., Rattanarat P., Chailapakul O. (2011). Nanoparticle-based electrochemical detection in conventional and miniaturized systems and their bioanalytical applications: A review. Analy. Chim. Acta.

[34] Liu T., Zhong J., Gan X., Fan C.H., Li G.X., Matsuda N. (2003). Wiring electrons of cytochrome c with silver nanoparticles in layered films. Chem. Phys. Chem..

[35] Xiao Y. (2003). Plugging into enzymes: Nanowiring of redox enzymes by a gold nanoparticle. Science.

[36] Xu J.Z., Zhu J.J., Wang H., Chen H.Y. (2003). Nano-sized copper oxide modified carbon paste electrodes as an amperometric sensor for amikacin. Analy. Lett..

[37] Zen J.M., Hsu C.T., Kumar A.S., Lyuu H.J., Lin K.Y. (2004). Amino acid analysis using disposable copper nanoparticle plated electrodes. Analyst.

[38] Kim G.Y., Shim J., Kang M.S., Moon S.H. (2008). Preparation of a highly sensitive enzyme electrode using gold nanoparticles for measurement of pesticides at the ppt level. J. Environ. Monitor..

[39] Box G.E.P. (2008). Estadística para Investigadores: Diseño, Innovación y Descubrimiento.

[40] Lewis G.A., Mathieu D., Phan R.T.L. (1999). Pharmaceutical Experimental Design.

[41] Massart D.L., Vandeginste B.G.M., Deming S.M., Michotte Y., Kaufman L. (2003). Chemometrics: A Textbook.

[42] Turdean G.L. (2011). Design and development of biosensors for the detection of heavy metal toxicity. Inter. J. Electrochem..

[43] Del Torno-de Román L., Alonso-Lomillo M.A., Domínguez-Renedo O., Arcos-Martínez M.J. (2013). Gluconic acid determination in wine by electrochemical biosensing. Sens. Actuators B Chem..

[44] Domínguez Renedo O., Arcos Martínez M.J. (2007). Anodic stripping voltammetry of antimony using gold nanoparticle-modified carbon screen-printed electrodes. Analy. Chim. Acta.

